# Insight to Psychological Aspects of Cancer

**DOI:** 10.1192/bjo.2022.206

**Published:** 2022-06-20

**Authors:** Ashkar Ali Kunhikkandy

**Affiliations:** Zaporozhye State Medical University, Zaporizhyia, Ukraine. IGNOU, Delhi, India

## Abstract

**Aims:**

The baseline of this study

1. What is the type of psychiatric disturbances in oncology settings?
2. Is there any importance in cancer education?
3. How to manage psychiatric disturbances?

**Methods:**

As of lockdown concerning COVID-19, this study is conducted online among 20 cancer patients. This is a cross-sectional study where Each patient has explained the purpose of the study, procedures, and consent was taken from patients then a questionnaire was given, and this was assessed. Among the profile of the study population, 50% were males and 50% were females of the total study population, 60% were married and 40% were unmarried, Participants were aged between 22 and 63 years. The study population also consists of 20% are breast cancer, 25% lung cancer,10% lung cancer, and the rest are other types of cancers. Patient details are collected from the Facebook groups for cancer patients. Assessment has 2 parts, one is based on CES-D Test where each individual was each patient answered 20 question and next part is based on 5 questions regarding Financial Depression, Behavioral changes, Feelings, Education about cancer and Psychiatric support.

**Results:**

It is found that 60% population are normal, 25% had mild Depression, 10% have moderate Depression followed by 5% with severe depression.

Among associations between marital status and various disorders, it was found that psychological disturbances are 2 times fold more in married people while compared to unmarried. There is also an association between treatment modalities are observed, in that anxiety is prevalent with people who had chemotherapy. Based on education and financial status, those who are with less education about cancer and less financially stable have also prominent disturbances.

**Conclusion:**

The study was based on other research study related to the spectrum of psychological disturbance based on treatment stage, financial status, awareness of cancer among patients, and role of marital status among individuals Offering mental health services to patients with cancer is becoming an integral part of oncologic treatments because psychological problems harm cancer management. The most common psychiatric disorders in cancer patients are depression, anxiety disorders, and adjustment disorders. Psychiatrists should be involved in the multidisciplinary treatment team that works with cancer patients. Further research is needed to determine the effectiveness of different psychological and psychopharmacological interventions in psycho-oncology and palliative medicine

